# Pathway of Programmed Cell Death and Oxidative Stress Induced by β-Hydroxybutyrate in Dairy Cow Abomasum Smooth Muscle Cells and in Mouse Gastric Smooth Muscle

**DOI:** 10.1371/journal.pone.0096775

**Published:** 2014-05-06

**Authors:** Wulin Tian, Teng Wei, Bin Li, Zhe Wang, Naisheng Zhang, Guanghong Xie

**Affiliations:** College of Veterinary Medicine, Jilin University, Changchun, China; University of Pecs Medical School, Hungary

## Abstract

The administration of exogenous β-hydroxybutyrate (β-HB), as well as fasting and caloric restriction, is a condition associated with β-HB abundance and decreased appetite in animals. Increased β-HB and decreased appetite exist simultaneously in some diseases, such as bovine left displaced abomasums (LDA) and human chronic gastritis. However, the effects of β-HB on stomach injuries have not been explored. To elucidate the possible effects of exogenous β-HB on the stomach, mice were injected intraperitoneally with β-HB, and bovine abomasum smooth muscle cells (BSMCs) were treated with different concentrations of β-HB. We found that β-HB induced BSMCs endoplasmic reticulum- and mitochondria-mediated apoptotic cell death. β-HB promoted Bax expression and caspase-12, -9, and -3 activation while blocking Bcl-2 expression. β-HB also promoted AIF, EndoG release and p53 expression. β-HB acted on key molecules in the apoptotic cell death pathway and increased p38 and c-June NH2-terminal kinase phosphorylation while inhibiting ERK phosphorylation and PCNA expression. β-HB upregulated P27 and P21 mRNA levels while downregulating cyclin and CDK mRNA levels, arresting the cell cycle. These results suggest that BSMCs treated with β-HB can induce oxidative stress, which can be prevented by intracellular calcium chelators BAPTA/AM but not antioxidant NAC. Additionally, these results suggest that β-HB causes ROS generation through a Ca^2+^-dependent mechanism and that intracellular Ca^2+^ levels play a critical role in β-HB -induced apoptotic cell death. The impact of β-HB on programmed cell death and oxidative stress *in vivo* was confirmed in murine experiments. For the first time, we show oxidative stress effects of β-HB on smooth muscle. We propose that β-HB is a possible cause of some stomach diseases, including bovine LDA.

## Introduction

DL-β-hydroxybutyrate (β-HB) is produced by the liver and used as an energy source by the brain, skeletal muscles and heart when glucose is not readily available. Generally β-HB level is maintained at a low level in serum. For example, the postprandial level of β-HB is approximately 0.05 mM in humans [3]. Increased β-HB levels are observed in some pathological physiological states, such as LDA in cows: between 1 and 7 d postpartum, increasing serum concentrations of β-HB have been associated with increased risk of subsequent LDA [4]–[6], and postpartum serum β-HB was a more sensitive and specific indicator of LDA than NEFA concentration. The odds of LDA were 8 times greater in cows with serum β-HB ≥1200 µmol/L [4]. Increased β-HB serum levels have also been found in the blood of pregnant women and humans with chronic gastritis [2], diabetic ketoacidosis, alcoholic ketoacidosis, salicylate poisoning and other rare conditions [7], [8].

Most investigators agree that a normal serum level of β-HB is less than 0.5 mM; a level greater than 1.0 mM can be defined as hyperketonemia, and a level greater than 3.0 mM can be defined as ketoacidosis [9]. Several studies have found that parenteral administration of β-HB in pygmy goats suppresses feed intake, while subcutaneously injected β-HB (10 mmol/kg body weight) significantly reduced feeding in rats [10]. In humans, postprandial β-HB serum concentration may rise to 1–2 mM after 2–3 days of fasting and reach 6–8 mM with prolonged starvation accompanied by loss of appetite [11], [12]. In these situations, the constriction function of the stomach is impaired to different degrees.

Gastric slow expansion is the pathological basis of bovine LDA. However, the role of β-HB in the pathogenesis of any stomach disease is less well known. Hence, we study the pro-apoptotic effect of β-HB on gastric smooth muscle cells. This study provides a novel understanding of β-HB and describes the role of β-HB in some stomach diseases such as bovine LDA.

## Results

### 2.1. Effects of β-HB on Cell Death

**Figure 1:**
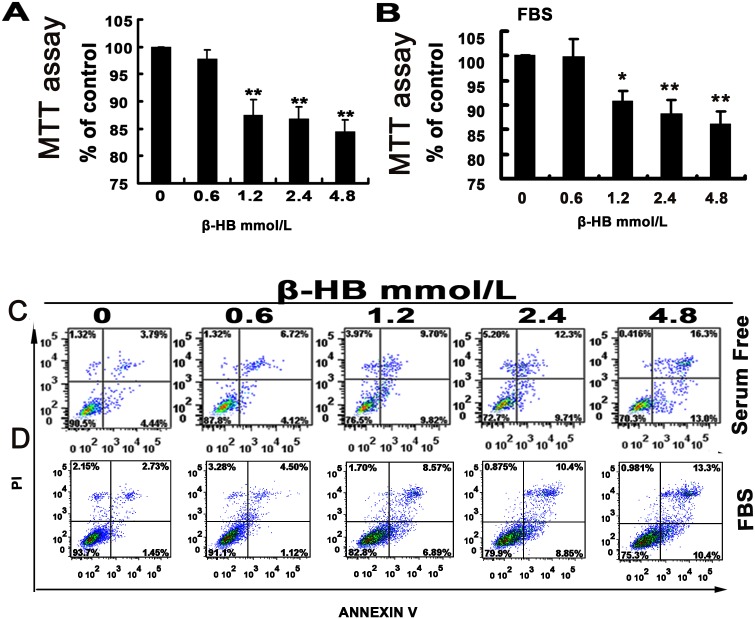
Effect of β-HB on cell death. Cells were incubated 48β-HB (0, 0.6, 1.2, 2.4 or 4.8 mM) in serum-free medium (A and C) or medium with 10% FBS (B and D). A) and B) Cell viability was determined with a MTT assay. Viability was calculated as the percentage of living cells in treated cultures compared to those in control cultures. C) and D) Mortality analysis. The mortality of BSMCs were assayed for with annexin V-FITC by flow cytometry. The mean of three independent experiments is shown. *p<0.05, **p<0.01 versus control group.

To evaluate cell viability, BSMCs were plated in 96-well plates and treated with different concentrations of β-HB in serum-free medium (as shown in Fig. 1A) and in medium with 10% FBS (as shown in Fig. 1B) for 48 h. Compared to the control group (0 mM β-HB), cell viability was significantly decreased in the 1.2, 2.4, 4.8 mM groups.

Next, we compared flow cytometry data to the control cell group. The cell groups in serum-free medium treated with different concentrations of β-HB (0, 0.6, 1.2, 2.4, 4.8 mM) indicated increased cell mortalities from 8.23% to 10.84%, 19.52%, 20.01% and 29.30%, respectively, as shown in Fig. 1C. Treatment of BSMCs with different concentrations of β-HB (0, 0.6, 1.2, 2.4, 4.8 mM) in medium with 10% FBS increased cell mortalities from 4.18% to 5.62%, 15.46%, 19.25% and 23.7%, respectively, as shown in Fig. 1D.

### 2.2. Effects of β-HB on the Glutathione Levels

GSH levels were measured in BSMCs cultured with different concentrations of β-HB, as shown in Table 1. BSMCs treated by β-HB in serum-free medium exhibited decreased GSH levels in a β-HB concentration-dependent manner, and the 2.4 mM β-HB group exhibited significantly decreased GSH levels compared to the control group (0 mM β-HB).

**Table 1:** Effects of β-HB on lipid peroxidation, glutathione level and antioxidant enzyme activities in BSMCs.

β-HB (mM)	MDA (µmol/mg protein)	CAT (U/mg protein)	SOD (U/mg protein)	GSH (mU/mg protein)
0	13.727±1.891	2.543±0.019	2.614±0.052	17.269±0.311
0.6	31.011±9.779*	2.307±0.052	2.389±0.046	16.517±1.789
1.2	55.561±4.403**	2.149±0.068*	2.091±0.054**	14.684±0.026
2.4	80.111±7.847**	1.418±0.186**	1.739±0.045**	10.559±0.622**

Cells were incubated 24 h with β-HB (0, 0.6, 1.2, 2.4 or 4.8 mM) in serum-free medium. The levels of MDA and GSH, as well as SOD and CAT activities were assayed by the methods as shown in 5.10–5.13. The mean of three independent experiments is shown.

*p<0.05,

**p<0.01 versus control group.

### 2.3. Effects of β-HB on Cellular Redox Status

BMSCs treated with β-HB in serum-free medium caused a concentration-dependent decrease in the activities of SOD and CAT, as shown in Table 1. SOD and CAT activities for the 1.2 and 2.4 mM β-HB groups were significantly decreased compared to the control group (BCG, 0 mM β-HB). In addition, treatment of BSMCs with 0.6, 1.2, 2.4 mM β-HB significantly increased intracellular MDA levels.

### 2.4. Effect of β-HB on Intracellular Calcium Level

**Figure 2:**
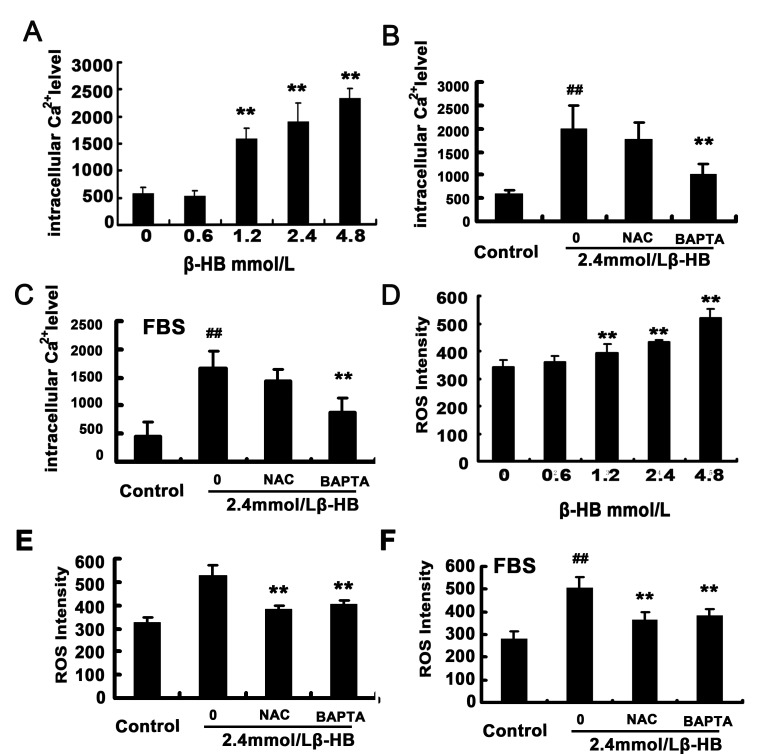
Effect of β-HB on intracellular ROS level and intracellular calcium level in BSMCs. Intracellular ROS level was measured with DCFH-DA by confocal microscopy. Intracellular calcium level was measured with Fura-3/AM by confocal microscopy. A) and D) Cells were incubated 12 h with β-HB (0, 0.6, 1.2, 2.4 or 4.8 mM) in serum-free medium. B) and E) Cells were incubated 12 h with β-HB (0, 2.4 mM) in serum-free medium with or without pretreatment with NAC (1 mM) or BAPTA/AM (15 µM). C) and F) Cells were incubated 12 h with β-HB (0, 2.4 mM) in medium with 10% FBS with or without pretreatment with NAC (1 mM) or BAPTA/AM (15 µM). The mean of three independent experiments is shown. *p<0.05, **p<0.01 versus control group.

Intracellular calcium levels were measured using a calcium indicator, Fura-3/AM. As shown in Fig. 2A, treatment of BSMCs with (1.2, 2.4, 4.8 mM) β-HB significantly elevated intracellular calcium levels 2.76, 3.22, 4.08-fold, respectively. A β-HB-induced increase of intracellular calcium levels was prevented by intracellular calcium chelators (BAPTA/AM) but not antioxidant NAC in serum-free medium (Fig. 2B) and in medium with 10% FBS (Fig. 2C).

### 2.5. Effect of β-HB on Intracellular ROS Generation

Overproduction of ROS plays a central role in oxidative stress. Intracellular ROS levels were detected with DCFH-DA by confocal microscopy. As shown in Fig. 2D, compared to the control group, β-HB treatment (1.2, 2.4, 4.8 mM) dose-dependently increased intracellular ROS generation in BSMCs. However, a β-HB-induced increase of intracellular ROS was prevented by intracellular calcium chelators (BAPTA/AM) and antioxidant NAC in serum-free medium (Fig. 2E) and in medium with 10% FBS (Fig. 2F).

### 2.6. Effects of β-HB on MAPKs Phosphorylation

**Figure 3:**
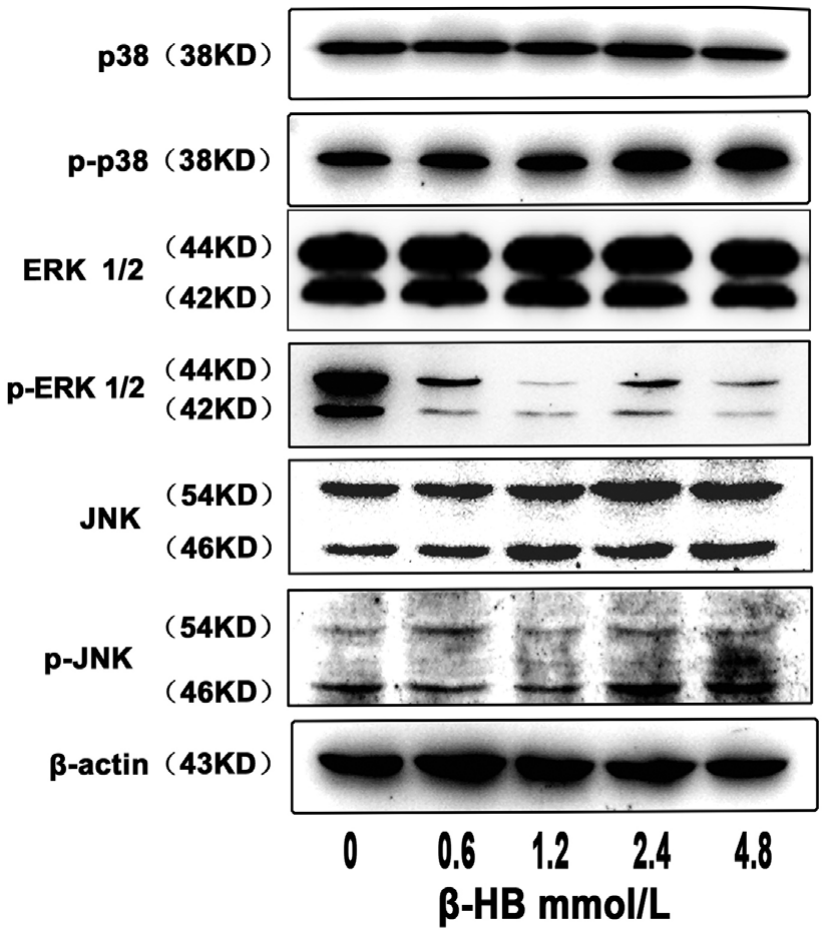
Effect of β-HB on phosphorylation of MAPKs in BSMCs. Cells were incubated 48β-HB (0, 0.6, 1.2, 2.4 or 4.8 mM) in serum-free medium. Phosphorylated-p38 and total p38, phosphorylated-JNK and total JNK, phosphorylated-ERK1/2 and total ERK1/2 were detected by Western blot.

We examined the phosphorylation of ERK1/2, p38 MAPK and JNK induced by β-HB using Western blot analysis. As shown in Fig. 3, phosphorylation of p38 was increased significantly in BSMCs treated with 2.4 and 4.8 mM β-HB, while p-JNK was increased significantly at β-HB concentrations of 0.6, 1.2, 2.4 or 4.8 mM. Phosphorylation of ERK1/2 was decreased in BSMCs treated with 0.6, 1.2, 2.4 or 4.8 mM β-HB. No changes in the expression of non-phosphorylated ERK, JNK and p38 kinase were observed in BSMCs treated with β-HB.

### 2.7. Effect of β-HB on p53 Activation

**Figure 4:**
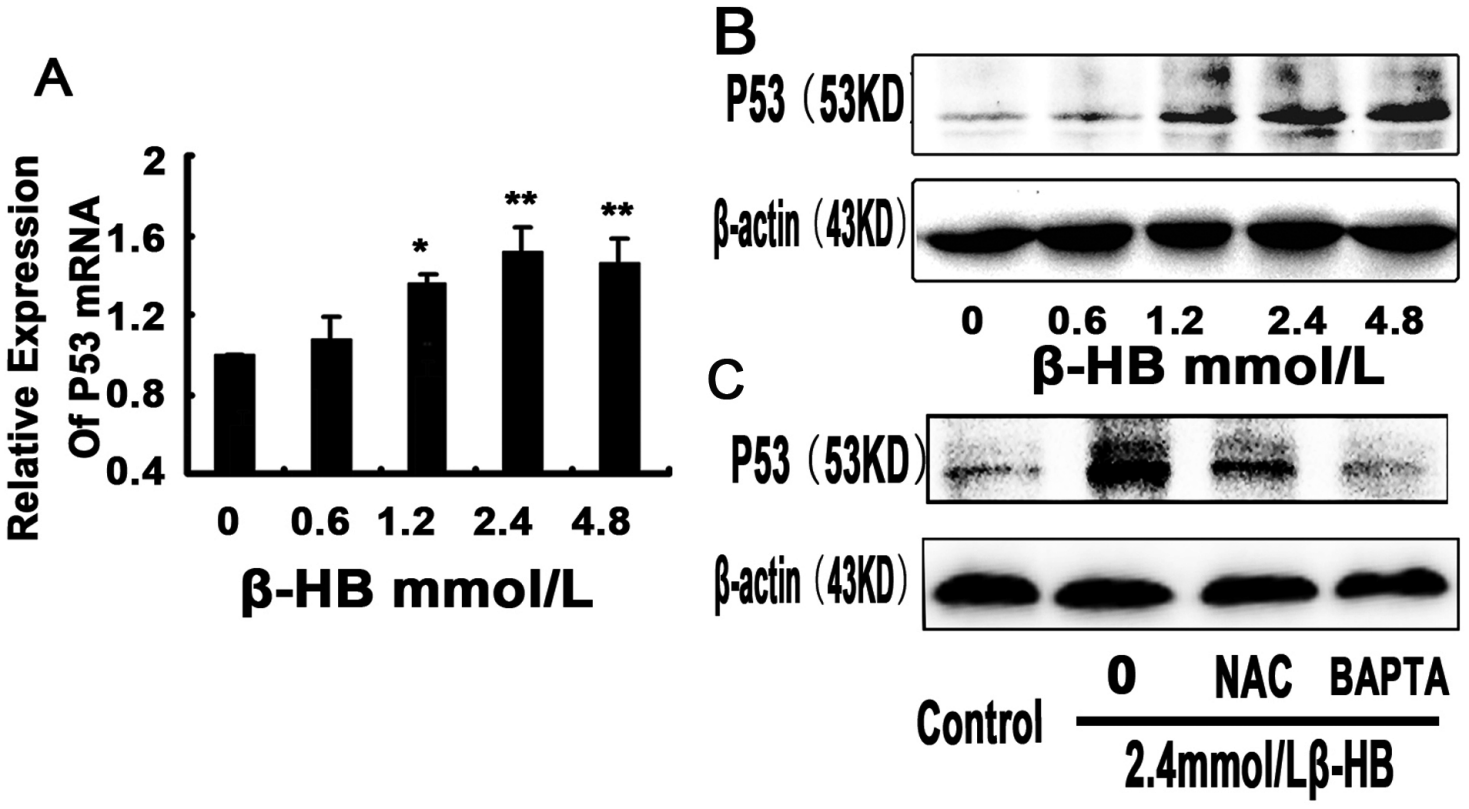
Effect of β-HB on the expression of p53 in BSMCs. Cells were incubated 48β-HB (0, 0.6, 1.2, 2.4 or 4.8 mM) in serum-free medium. A) p53 mRNA was detected by Q-PCR, and the ratio of p53 to β-actin was calculated. B) p53 protein was detected by Western blot, and the ratio of p53 to β-actin was calculated. C) Cells were incubated 48 h with β-HB (0, 2.4 mM) in serum-free medium with or without pretreatment with NAC (1 mM) or BAPTA/AM (15 µM). The mean of three independent experiments is shown. *p<0.05, **p<0.01 versus control group.

Because p53 plays a very important role in arresting the cell cycle and apoptosis, we measured the expression characteristics of p53 by RT-PCR and Western blot analysis in β-HB-treated BSMCs. As shown in Fig. 4, treatment with β-HB (1.2, 2.4 or 4.8 mM) upregulated mRNA and protein levels of p53. However, β-HB-induced p53 activation was prevented by intracellular calcium chelators (BAPTA/AM) or antioxidant NAC.

### 2.8. Effect of β-HB on Caspases Activation

**Figure 5:**
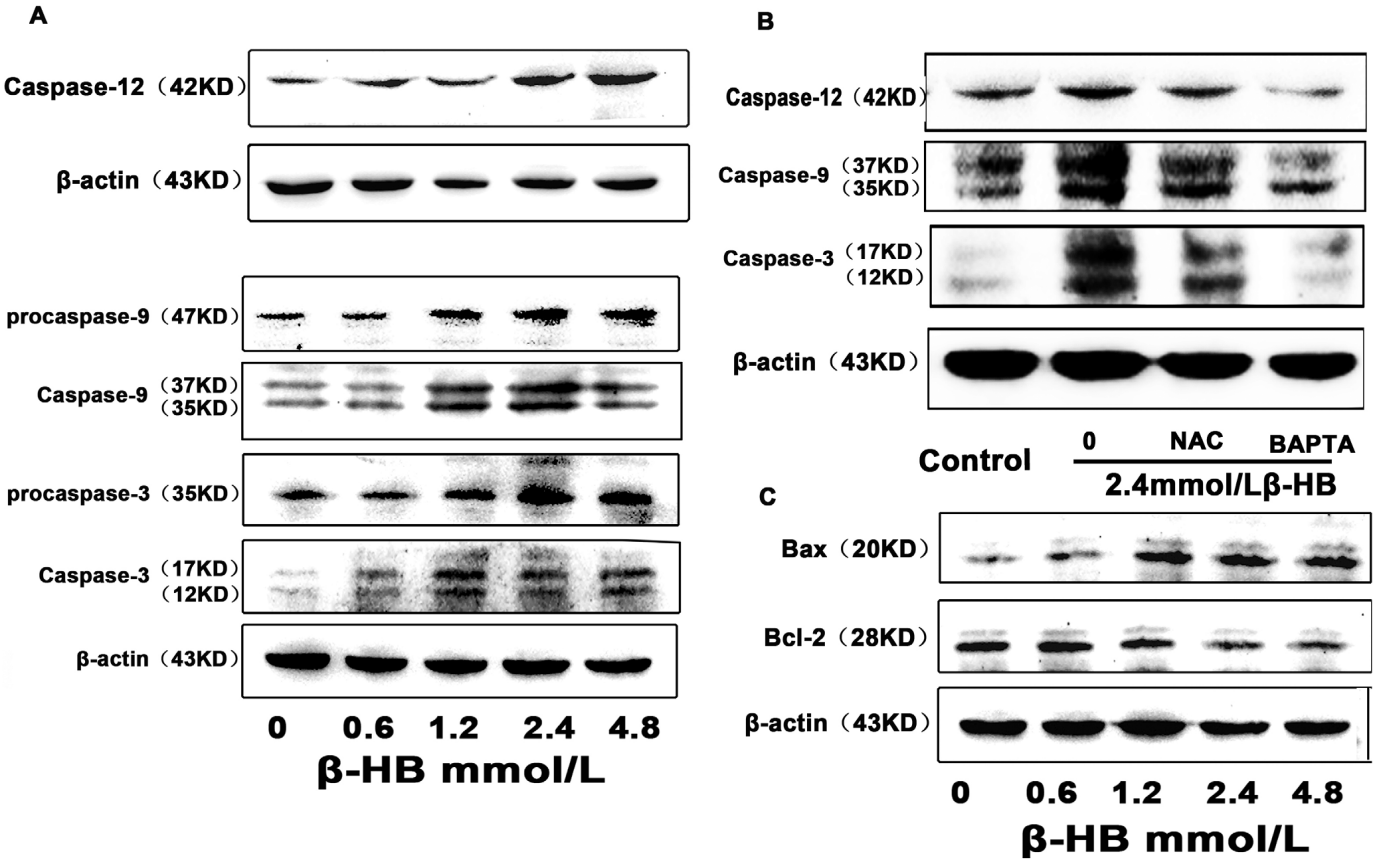
Effect of β-HB on the expression of cleaved caspase-12, -9, -3 Bax, Bcl-2 in BSMCs. A) and B) Cells were incubated 48 h with β-HB (0, 0.6, 1.2, 2.4 or 4.8 mM) in serum-free medium. Cleaved caspase-12, **-**9, **-**3, Bax and Bcl-2 were detected by Western blot. C) Cells were incubated 48 h with β-HB (0, 2.4 mM) in serum-free medium in the absence or presence of NAC (1 mM) or BAPTA/AM (15 µM). Cleaved caspase-12, **-**9, **-**3 were detected by Western blot.

Cleaved caspase-12, caspase-9 and caspase-3 were detected by Western blotting. As shown in Fig. 5AB, compared to the control group, β-HB treatment (1.2, 2.4, 4.8 mM) significantly induced caspase-12, caspase-9 and caspase-3 activation in BSMCs, while activation was prevented by intracellular calcium chelators (BAPTA/AM) or antioxidant NAC.

### 2.9. Effects of β-HB on Bax and Bcl-2 Expression

To investigate the effects of β-HB on Bax and Bcl-2, two proteins in the caspase-dependent apoptotic pathway, Bax and Bcl-2 were detected by Western blotting. As shown in Fig. 5C, BSMCs with β-HB exhibited an increased ratio of Bax to Bcl-2 in a dose-dependent manner.

### 2.10. Effect of β-HB on AIF and EndoG Expression and Translocation

**Figure 6:**
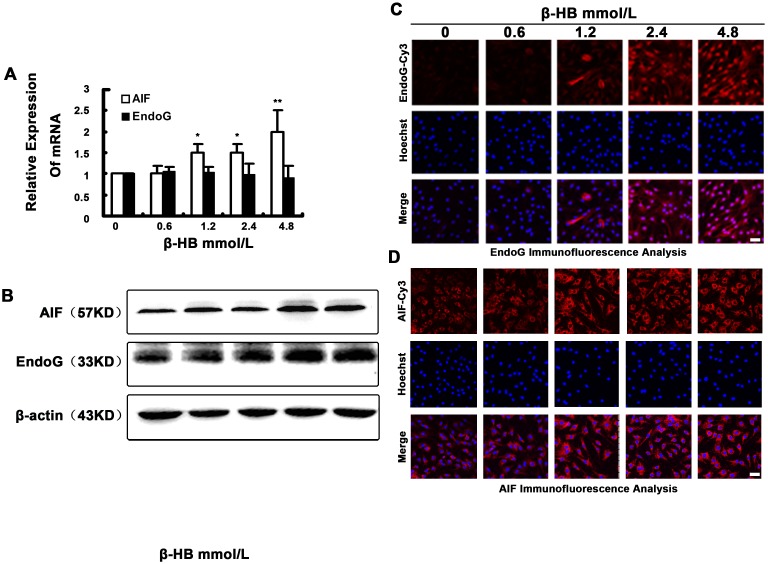
Effect of β-HB on AIF and EndoG expression and translocation in BSMCs. Cells were incubated 48β-HB (0, 0.6, 1.2, 2.4 or 4.8 mM) in serum-free medium. A) AIF and EndoG mRNA were detected by Q-PCR, and the ratios of AIF and EndoG to β-actin were calculated. B) AIF and EndoG protein were detected by Western blot. C) and D) Protein localization of AIF and EndoG was detected by immunofluorescence. The mean of three independent experiments is shown. *p<0.05, **p<0.01 versus control group.

We determined the expression levels of AIF and EndoG in BSMCs by RT-PCR. In addition, AIF and EndoG were detected by Western blot analysis using antibodies against AIF and EndoG. We observed a significant rise in AIF and EndoG mRNA levels in the 2.4 mM group and 4.8 mM group (Fig. 6A). AIF and EndoG were expressed at significantly higher levels in the 1.2, 2.4 and 4.8 mM groups (Fig. 6B).

To further determine whether EndoG (or AIF) leads to the BSMCs death, we investigated EndoG (or AIF) translocation from the mitochondria into the nucleus during β-HB-induced apoptotic cell death. As shown in Fig. 6CD, subcellular fractionation and confocal microscopy clearly showed that the treatment with β-HB (1.2, 2.4 or 4.8 mM) significantly increased AIF and EndoG release. EndoG was translocated from the mitochondria into the nucleus, but AIF was observed around the nucleus following treatment with β-HB in BSMCs.

### 2.11. Effect of β-HB on PCNA Induction

**Figure 7:**
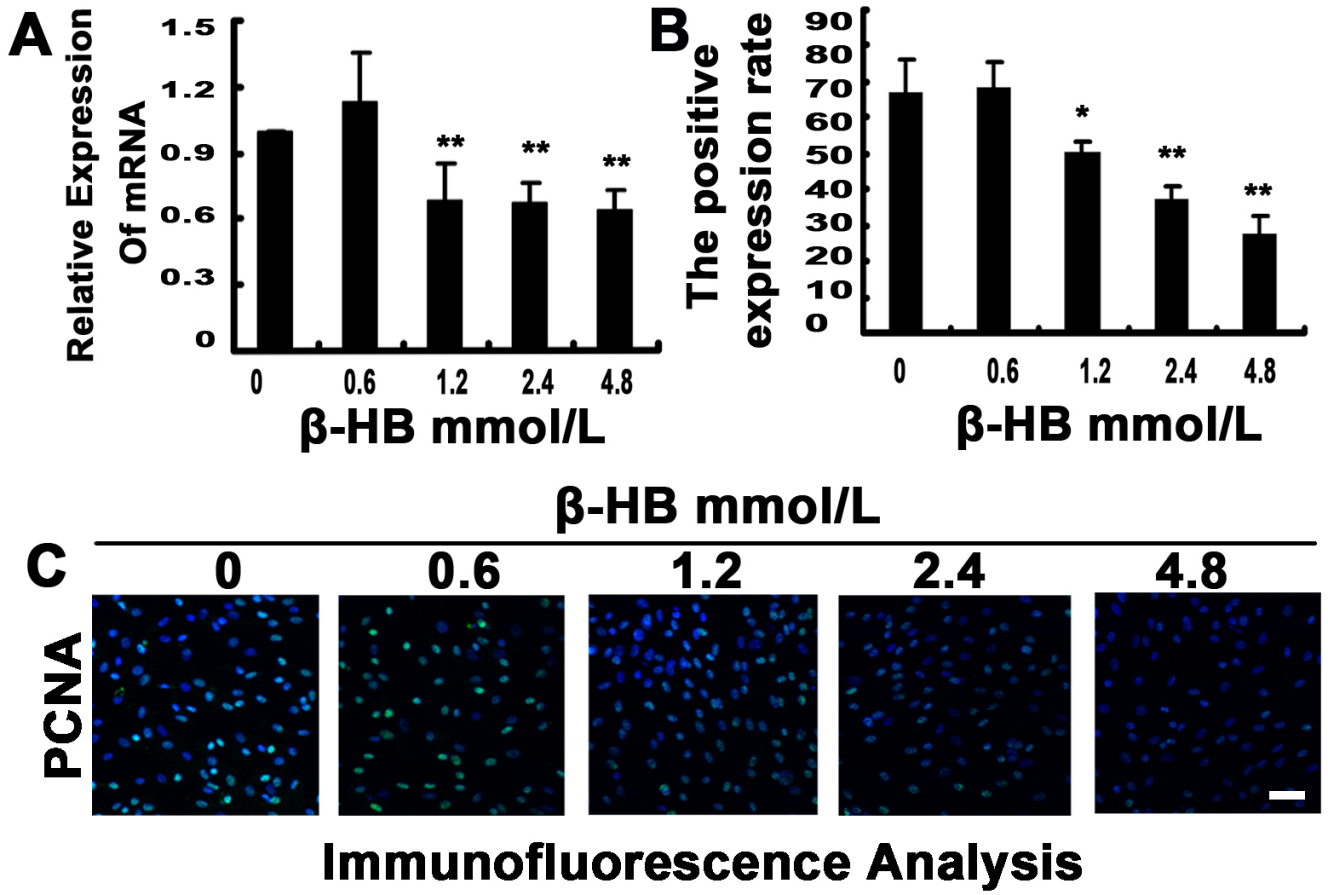
Effect of β-HB on PCNA induction. Cells were incubated 48β-HB (0, 0.6, 1.2, 2.4 or 4.8 mM) in serum-free medium. A) PCNA mRNA was detected by Q-PCR, and the ratio of PCNA to β-actin was calculated. B) The percentage of PCNA was measured from the immunofluorescence in figure 7C. C) Protein localization of PCNA was detected by immunofluorescence. The mean of three independent experiments is shown. *p<0.05, **p<0.01 versus the control group.

Proliferating cell nuclear antigen (PCNA) is a DNA polymerase processing protein and is essential for DNA replication in many cell types. As shown in Fig. 7, we observed a significant reduction in PCNA mRNA levels in β-HB-treated BSMCs in the 1.2, 2.4 and 4.8 mM groups (Fig. 7A). β-HB treatment significantly decreased the expression of PCNA in BSMCs as determined via immunofluorescence for 24 h (Fig. 7B and C).

### 2.12. Effect of β-HB on Cell Cycle

**Figure 8:**
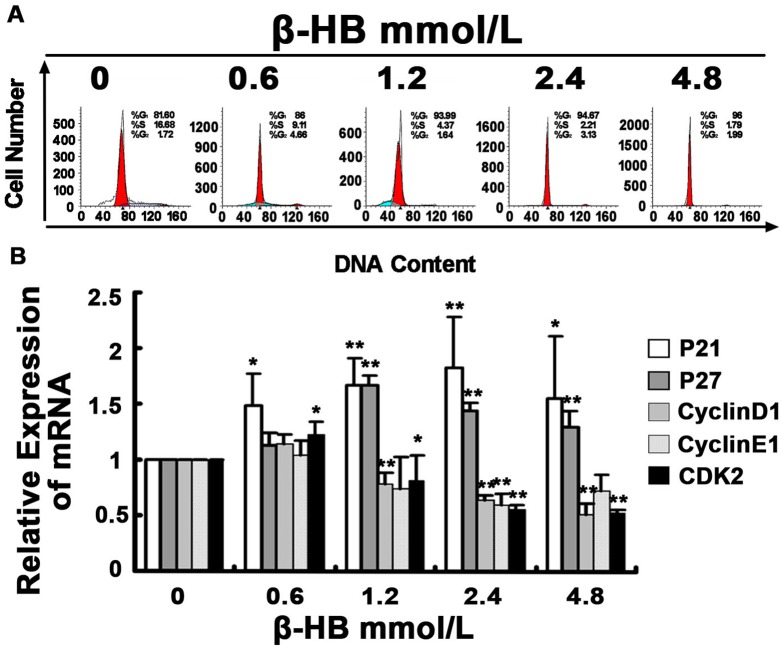
Effect of β-HB on cell cycle. Cells were incubated 48β-HB (0, 0.6, 1.2, 2.4 or 4.8 mM) in serum-free medium. A) Cell cycle analysis was performed by flow cytometry to determine the percentage of cells in the G1, S, and G2 phases. B) The mRNAs of P21, P27, cyclin D1, CDK2 and cyclin E1 were detected by Q-PCR, and the ratios of these mRNA levels to β-actin were calculated. The mean of three independent experiments is shown. *p<0.05, **p<0.01 versus control group.

Cell cycle changes were analyzed by flow cytometry to determine the effect of β-HB. As shown in Fig. 8A, the ability of BSMCs to begin mitosis, progress through G1, and enter S phase was evaluated by measuring the DNA content in BSMCs with various concentrations of β-HB. Approximately 81.6% of the cells were in G1 phase and 16.7% of the cells were in S phase in the control group. We observed a significant increase of cells in G1 phase in β-HB-induced BSMCs in the 1.2, 2.4 and 4.8 mM groups, while a significant decrease of cells in S phase was observed in these groups. These results indicated that β-HB strongly induced cell cycle arrest in the G1 phase.

### 2.13. Effect of β-HB on the mRNA Levels of p27, p21, Cyclins and CDKs

The mRNA expression levels of p27, p21, CDK1, CDK2 and cyclin E1 were measured by quantitative RT-PCR. As shown in Fig. 8B, compared with the control group, there was a significant increase (P<0.05) in the P27 and P21 mRNA levels in the 1.2, 2.4 and 4.8 mM groups, while mRNA levels of CDK1 and CDK2 were significantly decreased in these groups. mRNA levels of cyclin E1 were significantly decreased in the 2.4 mM group and slightly decreased in the 1.2 and 4.8 mM groups. However, there was a slight increase of CDK1, CDK2 and cyclin E1 mRNA levels in the 0.6 mM group.

### 2.14. Effects of β-HB on Apoptosis *In vivo*

**Figure 9:**
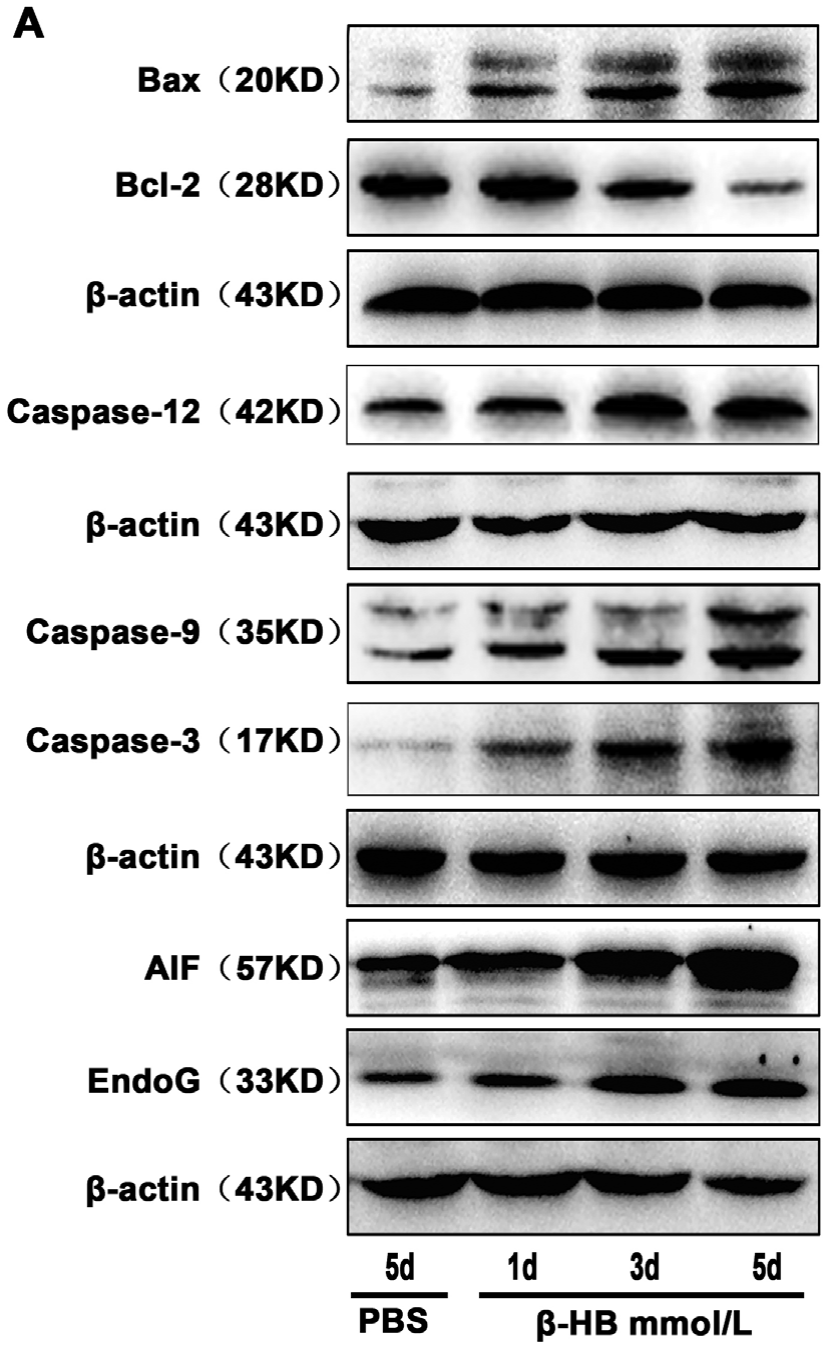
Effects of β-HB on the expression of apoptosis proteins *in vivo*. Mice were intraperitoneally given β-HB or PBS (control). Cleaved caspase-12, **-**9, **-**3, Bax, Bcl-2, AIF and EndoG protein were detected by Western blot.

To confirm the effectiveness of β-HB in vivo, we investigated the protein expression of several apoptosis-related proteins in gastric smooth muscle of β-HB injected mice as well as control mice. As shown in Fig. 9, compared to the control, we found that β-HB treatment increased the expression of caspase-12, -9, -3, Bax, AIF and EndoG, while blocking Bcl-2 expression. These results were consistent with results in BSMCs.

## Discussion

β-HB is an important intermediate of lipid metabolism, but the physiological role of β-HB is less well known. It seems that β-HB has different effects on different cell types. β-HB shows protective effects on HEK293 cells [1] and PC12 cells [13]. On the other hand, β-HB is also able to induce oxidative stress on adult cardiomyocytes [14] and growth inhibition in HK-2 [15] and SK-N-AS neuroblastoma cells [16]. Among the several cell types studied, we found that BSMCs were sensitive to β-HB (from 1.2 mM), so BSMCs were used to investigate the mechanism of β-HB-induced apoptosis. Investigation of this mechanism may allow for the elucidation of the pathogenesis of LDA, which is often indicated by postpartum serum β-HB in cows: increased β-HB levels were associated with increased risk of subsequent LDA.

We performed a MTT assay to study the effect of β-HB on BSMC viability. Cell viability was significantly decreased in the β-HB-treated groups in both serum-free medium and medium with 10% FBS, suggesting that β-HB had cytotoxic effects and induced cell death. To confirm whether the type of cell death is apoptosis or necrosis, a flow cytometric analysis of apoptosis was performed, finding that β-HB indeed induced apoptotic cell death in both serum-free medium and medium with 10% FBS.

Oxidative stress initiates apoptosis in many cell types [17], [18]. From our study, we found that β-HB treatment increased ROS production. It was confirmed that β-HB-induced BSMCs apoptosis is mediated by activation of oxidative stress-responsive cell signaling. Oxidative stress changes the enzymatic and nonenzymatic antioxidant defense [19]. The activities of the antioxidant enzymes SOD and CAT as well as the levels of the non-enzymatic antioxidant molecules GSH and lipid peroxidation (MDA as an index) were assayed in the present study. It was found that β-HB significantly decreased the activities of the antioxidant enzymes and GSH levels while increasing lipid peroxidation. These results showed that oxidative stress is involved in β-HB-induced apoptosis.

Elevated intracellular Ca^2+^ levels are involved in the apoptotic cell death mechanisms of various cells [20], [21]. The ER is a major intracellular Ca^2+^ storage compartment, playing a critical role in maintaining cellular Ca^2+^ homeostasis [22]. Enhanced Ca^2+^ concentrations, along with cleavage of procaspase-12, which results in increased level of cleaved caspase-12, are known major elements of ER stress that lead to caspase-dependent apoptotic cell death. From our study, β-HB exposure caused an increase in intracellular Ca^2+^ levels and expression of cleaved caspase-12. However, the correlation between intracellular Ca^2+^ and ROS generation is complex. According to some reports, ROS can induce an increase in intracellular Ca^2+^ levels [23], [24]. On the other hand, increased intracellular Ca^2+^ can promote ROS generation [25], [26]. Accumulation of intracellular Ca^2+^ induces formation of free radicals and increases production of ROS by the mitochondrial respiratory chain [27]. In present study, intracellular Ca^2+^ levels were not affected by NAC, while β-HB -induced increases in ROS generation were blocked by pretreatment with BAPTA/AM in both serum-free medium and medium with 10% FBS. These results suggest that β-HB causes ROS generation through a Ca^2+^-dependent mechanism and that intracellular Ca^2+^ levels play a critical role in β-HB -induced apoptotic cell death in both serum-free medium and medium with 10% FBS.

The mammalian family of mitogen-activated protein kinases (MAPKs), including extracellular signal-regulated kinase (ERK), p38 and the c-Jun NH2-terminal kinase (JNK), which controls cell migration, proliferation, and differentiation, were activated following increased Ca^2+^ levels and oxidative stress (ROS production) [28], [29]. Increased level of activated (phosphorylated) JNK and p38 were consistently detected in β-HB-treated cells, and JNK and p38 are believed to phosphorylate various substrate proteins, such as p53. Once p53 is activated, it acts as a transcription factor for many genes containing the consensus p53-binding sites in their promoter or intronic sequences [30], inducing the expression of many proapoptotic proteins such as Bax, which in turn induces the permeabilization of the mitochondrial outer membrane [31]. It has been suggested that the transcription-independent functions of p53 cooperate with the transcription-dependent functions of p53 in the induction of apoptosis. Phosphorylated-p53 can directly bind and activate Bax [32]. Direct importing of p53 into the mitochondrial matrix upon ROS generation has also been proposed [33], [34]. In this study, consistent with activated MAPKS, activated p53 was observed in β-HB-treated group’.

According to our results, the ratio of Bax/Bcl2 showed a significant increase in β-HB-treated cells. It has been reported that either Bcl2 overexpression or the loss of Bax leads to a reduced resting ER Ca^2+^ level and a secondary decrease in Ca^2+^ uptake into mitochondria [35], [36]. Bax-mediated alterations in ER and mitochondrial Ca^2+^ levels are regarded as important upstream signals for cytochrome c (Cyt c) release, an essential factor in caspase-dependent apoptotic cell death [37], [38]. In addition to inducing mitochondrial membrane permeabilization (mmp) by Ca^2+^ released from the ER in response to ROS [39], an increased Bax/Bcl2 ratio can also directly induce mmp, leading to the release of Cyt c along with other proapoptotic proteins such as Apoptosis inducing factor (AIF) and endonuclease G (Endo G) [40]. AIF and Endo G are two mitochondrial proteins that trigger chromatin condensation and DNA degradation to induce programmed cell death [41]. These two proteins were found to be effectors of caspase-independent death that can allow independent nuclei to undergo apoptotic changes. Cell death starts with an alteration of mitochondrial membrane potential and the release of AIF and Endo G into the cell nucleus to condense chromosomes and fragment DNA molecules. In this study, we observed that the intranuclear fluorescence of AIF and Endo G in the β-HB-treated group was significantly enhanced compared to the control, suggesting that the mitochondrial caspase-independent pathway is also involved in β-HB-induced apoptotic cell death.

Once released, Cyt c forms an oligomeric complex with dATP and Apaf-1 [42], followed by recruitment and activation of procaspase-9. Active caspase-9 then activates effector caspases, such as caspase-3, -6 and -7 [43]. An increased level of cleaved caspase-3 was detected in β-HB-treated groups, and this increased level cannot be totally attributed to activation of the mitochondria-mediated apoptotic pathway upon β-HB exposure, as the expression of cleaved caspase-3 can also be induced following activation of the ER-mediated apoptotic pathway. An increased level of activated caspase-12, which was detected in β-HB -treated groups and is believed to be released from stressed ER, directly activates procaspase-9 and the downstream effector caspases, caspase-3 and -7, independently of cytochrome c and Apaf-1 [44].

Our findings in vivo and in vitro suggest that, following β-HB exposure, the major apoptotic pathway leading to the huge amount of observed cell death, is ER- and mitochondrial-mediated apoptotic cell death. Excessive Ca^2+^ influx is one of the major triggers of caspase overactivation [45] and had been reported to be involved in p53 activation [46]. β-HB exposure not only induced caspase-dependent apoptosis but also induced p53 activation, both of which were largely inhibited by pretreatment with the intracellular Ca^2+^ chelator BAPTA/AM. These results further confirm that intracellular Ca^2+^ levels play a critical role in β-HB-induced apoptotic cell death.

To investigate the depression of cell proliferation caused by β-HB, we investigated the effect of β-HB on PCNA and the cell cycle of BSMCs. PNCA was highly expressed in rapidly proliferating cell nuclei in a variety of tissues and cell lines and was identified as the 34 kDa processing factor of DNA polymerase delta. It has been reported that PCNA is involved in DNA damage and cell-cycle arrest via depression of P21, apoptosis, ATPases and protein kinases [47], [48]. In our experiment, β-HB reduced PCNA mRNA levels in a dose-dependent manner, and this result was consistent with the expression of PCNA protein as observed by immunofluorescence. Cell proliferation is involved in programmed cell death along with cell cycle arrest [49]. β-HB strongly induced cell cycle arrest in the G1 phase. It is believed that progression through the G1 phase of the cell-cycle is regulated by activation of the cyclins (cyclin A, D and E), which is associated with activation of cyclin-dependent kinases (CDK1, CDK2 and CDK4). These CDK-cyclin complexes are subject to negative regulation by cyclin-dependent kinase inhibitors (CDKIs) such as p21, p27 and p57. Up-regulation of CDKIs during late G1 after addition of growth inhibitors in vitro is a critical step in inhibiting activation of cyclin-CDK complexes and subsequent arresting of cell cycle in the G1 phase. Consistently, we also detected increased mRNA levels of P21 and P27, while mRNA expression levels of CDK1, CDK2 and cyclin E1 were depressed in β-HB-treated BSMCs. These results suggest that β-HB-induced cell death is also associated with cell cycle arrest.

## Conclusions

The present investigation provides strong evidence supporting the oxidative stress effects of β-HB both in vitro and in vivo. β-HB treatment induced ER- and mitochondrial mediated apoptotic cell death through increased intracellular calcium. β-HB treatment also arrested the cell cycle at the G1 phase through inactivation of cyclins/CDKs. Our study presents evidence for potential stomach injury as a result of high levels of blood β-HB.

## Materials and Methods

### Ethics Statement

Procedures were conducted according to the US NIH Guide for the Care and Use of Laboratory Animals and approved by the Animal Welfare and Research Ethics Committee at Jilin University (Approval ID: 20111210-3).

### 5.1. Materials

β-HB was obtained from Sigma Aldrich (St. Louis, MO, USA). Antibodies directed against caspase-12, phospho-p38 MAP kinase, phospho-SAPK/JNK, p38 MAP kinase, SAPK/JNK, Phospho-ERK1/2, ERK1/2 were obtained from Cell Signaling Technology (Danvers, MA, USA). AIF and ENDOG antibodies were obtained from Santa Cruz Biotechnology, Inc. (Dallas, TX, USA). Antibodies directed against β-actin were obtained from Sungene Biotech (Nanjing, Jiangsu, China). Antibodies directed against PCNA, caspase-9, caspase-3, bax and bcl-2 were obtained from Boster Biotechnology (Wuhan, Hubei, China). Antibody directed against p53 was obtained from Keygen Biotech (Nanjing, Jiangsu, China). Fura-3/AM and BAPTA/AM were obtained from Dojindo Laboratories (Mashikimachi, Japan). NAC was obtained from beyotime institute of biotechnology (Haimen, Jiangsu, China). All the other reagents, unless otherwise stated, were purchased from Sigma Aldrich (St. Louis, MO, USA).

### 5.2. Cell Culture and Treatment

BSMCs of dairy calf (provided by the Baishan Co. Ltd., Jilin province, China.) were cultured using enzymatic digestion, as previously described by HU, W.Y, et al. BSMCs were cultured with Dulbecco’s modified Eagle medium (DMEM) (Thermo Scientific Hyclone, Logan, UT, USA) supplemented with 10% FBS (fetal bovine serum) and 100 U/ml of penicillin, in a humidified atmosphere at 37°C with 5% CO_2_ for 48 h.

Then, BSMCs were incubated with β-HB (0, 0.6, 1.2, 2.4 or 4.8 mM) in medium with or without 10% FBS for 48 h/24 h/12 h. Separately, BSMCs were pretreated 30 min with or without NAC (1.0 mM) or BAPTA/AM(15 µM) and incubated with β-HB (2.4 mM) in serum-free medium for 48 h/12 h.

### 5.3. Animals and Treatment

In total, twenty-four adult female Kun-ming mice, 4–5 weeks old, weighing 25–28 g were used. These mice were provided by the Center of Experimental Animals of Baiqiuen Medical College, Jilin University, China.

Mice were randomly divided into four groups (6 mice per group) as follows: control group (CG): these six mice were injected intraperitoneally with distilled water, 0.15 ml/mouse, six times at 24 h intervals from 0 to 5 days until sacrifice on the sixth day. The first, second and third groups were injected intraperitoneally with β-HB (1 M, diluted with distilled water), 0.15 ml/mouse, one time, three times and five times, respectively at 24 h intervals, then sacrificed 24 h after the last injection. Stomach tissues were rapidly excised and weighed, snap-frozen in liquid nitrogen and kept at −80°C for protein extraction.

### 5.4. Measurement of Cell Viability

BSMCs were incubated with β-HB (0, 0.6, 1.2, 2.4 or 4.8 mM) in medium with or without 10% FBS. Cell viability was determined by a MTT reduction assay. Briefly, dark blue formazan crystals formed in intact cells were solubilized in DMSO, and the absorbance of the resulting solution was measured at 570 nm. Results were expressed as the percentages of reduced MTT, assuming the absorbance of control cells to be 100%.

### 5.5. Measurement of Intracellular Calcium Level

BSMCs were seeded in 24-well tissue plates. After 24 h, BSMCs were treated with different concentrations of β-HB in medium with or without 10% FBS for 12 h with or without NAC (1.0 mM) or BAPTA/AM (15 µM). Every sample was washed in HEPES buffer solution twice. Fura-3/AM (final concentration 10 µM) was added to every sample in 24-well tissue plates, and samples were incubated at 37°C for 30 min. Every sample was washed in HEPES buffer solution twice. Fluorescence measurements were performed using an Olympus Fluoview-10 confocal microscope. Fluo-3 was excited at a wavelength of 488 nm, and fluorescence was measured at a wavelength of 515 nm. Image processing and analysis was performed by Olympus Fluoview software, and intracellular calcium level was expressed as the average fluorescence intensity.

### 5.6. Immunofluorescence Analysis

BSMCs were plated on sterile glass slides in 24-well tissue plates and incubated at 37°C for 48 h with β-HB (0, 0.6, 1.2, 2.4 or 4.8 mM) in serum-free medium. Slides were rinsed three times in phosphate-buffered saline (PBS, pH 7.4) and fixed with 10% neutral buffered formalin at room temperature for 20 min. Cells were rinsed in PBS and treated with 1 M EDTA-Na_2_ (pH = 9.0) for 5 min at 95°C prior to rinsing in PBS. Cell were then treated with 0.1% triton-100 for 10 min. After rinsing in PBS, cells were blocked with 5% normal donkey serum for 30 min in a humidified box and then incubated with anti-human AIF (1∶50), ENDOG (1∶50), and PCNA(1∶100) at 4°C overnight. The slides were rinsed three times in PBS and incubated with Cy3-conjugated donkey anti-goat immunoglobulin G and Alexa Fluor 488-conjugated donkey anti-mouse immunoglobulin G for 30 min. Finally, the cells were rinsed with PBS, stained with Hoechst dye and viewed under a Olympus Fluoview-10 confocal microscope.

### 5.7. Western Blotting Analysis

BSMCs were incubated 48 h with β-HB (0, 0.6, 1.2, 2.4 or 4.8 mM) in serum-free medium, then the cells were lysed in buffer(RIPA lysis buffer)containing complete protease inhibitor cocktail and phosphatase inhibitor mixture. The protein samples were separated by SDS-PAGE and electro-transferred onto polyvinylidene fluoride membranes (Millipore Corporation, Billerica, MA, USA). The blots were incubated with specific antibodies. After rinsing in TBST, the blots were incubated with appropriate secondary antibodies conjugated to horseradish peroxidase (HRP). Immunoreactive bands of proteins were detected by an enhanced chemiluminescence detection system, and the membrane was exposed to an X-ray film. Protein levels were normalized by blots against β-actin.

### 5.8. Flow Cytometric Analysis of Cell Cycle and Apoptosis

BSMCs treated with β-HB in medium with or without 10% FBS in six-well plates for 48 h were collected and washed with PBS. After resuspension in 800 µl PBS, 200 µl CyStain (Partec GmbH, Germany) was added for cell cycle experiments. An Annexin V-FITC apoptosis detection kit was used to detect PS expression on the cell membrane, an indicator of apoptotic cells. Briefly, the treated cells were harvested and washed with PBS twice, gently resuspended in annexin-V binding buffer, incubated with annexin V-FITC in the dark for 10 min and subsequently incubated with PI in the dark for 10 min. The treated cells were then analyzed by flow cytometry using Cell Quest software (BD Biosciences, USA). The results were expressed as percentages of cell population in different quadrants over total cells using quadrant statistics. “Dead” cells included apoptotic and necrotic cells or post-apoptotic necrotic cells. Cells in the lower right quadrant represented apoptosis and in the upper right quadrant represented necrosis or post-apoptotic necrosis.

### 5.9. Real-time Fluorescent Quantitative PCR and PCR Analysis

BSMCs were incubated 48 h with β-HB (0, 0.6, 1.2, 2.4 or 4.8 mM) in serum-free medium. The relative levels of AIF, ENDOG, P21, P27, CDK2, Cyclin D1, Cyclin E1, P53, and PCNA mRNA in BSMCs were examined by RT-PCR (SYBR Green) (ABI 7500, fluorescent quantitative PCR instrument; Applied Biosystems, USA). The BSMCs were treated with different concentrations of β-HB for 48 h. The gGene levels were normalized to the gene levels of β-actin. Specific primers for AIF, ENDOG, P21, P27, CDK2, Cyclin D1, Cyclin E1, P53, PCNA, and β-actin were designed by the Primer Premier Software (PREMIER Biosoft International, USA) based on known dairy cow sequences, as shown in Table 2.

**Table 2:** Primers used for quantitative real-time PCR.

Primers	Accession NO.	Forward	Reverse	bp
P21	NM001098958.2	TGCTGGGTGTTACGAAGTCC	CACCCTGCCCAACCTTAGAG	164
P27	NM001100346.1	AAACCCAGAGGACACGCATT	GTTGGGGGAACCGTCTGAAA	172
P53	NM001098958.2	ACACAACCTTCCAGGGAGCTA	GTGGCAACATCTGTGTACGG	164
CDK2	NM001014934.1	GCCACTCTCATCGAGTCCTG	CGGTACCACAGAGTCACCAC	154
CyclinE1	NM001192776.1	GCCACTGCCTGTACTGAACT	TTGCTCGCATTTTAGGCTGC	131
CyclinD1	NM001046273.2	CCTCTCCTATCACCGCCTGA	TTTGGGGTCCAAGTTCTGCT	142
PCNA	NM001034494.1	CCTTGGTGCAGCTAACCCTT	TACTAGTGCCAACGTGTCCG	170
AIF	NM001192884.1	ATGCTGTTGTGAGCGGAAGA	CTCTGTGGCAGATTTGGGGT	197
ENDOG	NM175823.3	CAAGTCATTGGCAAGAACC	CATCACATAGGAGCGGAGT	102
β-actin	NM173979.3	GCCCTGAGGCTCTCTTCCA	GCGGATGTCGACGTCACA	101

### 5.10. Measurement of Glutathione Peroxidase Levels

Cells were incubated 24 h with β-HB (0, 0.6, 1.2, 2.4 or 4.8 mM) in serum-free medium. The concentration of total glutathione peroxidase (GSH-Px) was determined in whole cell lysates using dithionitrobenzoic acid (DTNB) at 340 nm. One unit of glutathione peroxidase activity was defined as the amount required to oxidize 1 µM NADPH to NADP+ in 1 min at 25°C, pH 8.0. The concentration of total GSH was normalized by the standard substance of GSH.

### 5.11. Superoxide Dismutase Activity Assay

Cells were incubated 24 h with β-HB (0, 0.6, 1.2, 2.4 or 4.8 mM) in serum-free medium. Superoxide dismutase (SOD) activity was measured based on the extent of inhibition of amino blue tetrazolium formazan formation in a mixture of nicotinamide adenine dinucleotide, phenazine methosulphate and nitroblue tetrazolium (NADHPMS-NBT), according to the method of Kakkar et al. The color intensity of chromogen in butanol was measured by spectrophotometry at 560 nm. One unit of enzyme activity was defined as the amount of enzyme which caused 50% inhibition of NBT reduction/mg protein.

### 5.12. Catalase Activity Assay

Cells were incubated for 24 h with β-HB (0, 0.6, 1.2, 2.4 or 4.8 mM) in serum-free medium. BSMCs were treated with different concentrations of β-HB for 24 h. Catalase (CAT) activity in the culture medium and cell extract were measured with a Catalase Assay Kit according to the manufacturer’s protocol. Briefly, samples were treated with excess hydrogen peroxide for decomposition by catalase for a set time, and the remaining hydrogen peroxide coupled with a substrate was treated with peroxidase to generate a red product, N-4-antipyryl-3-chloro-5-sulfonate-p-benzoquinonemonoimine, which absorbs maximally at 520 nm.

### 5.13. Measurement of Lipid Peroxidation

Cells were incubated 24 h with β-HB (0, 0.6, 1.2, 2.4 or 4.8 mM) in serum-free medium. Malondialdehyde (MDA) levels were measured with a Lipid Peroxidation MDA Assay Kit according to the manufacturer’s protocol. We used a protein assay kit (Bio-Rad Laboratories, Hercules, CA, USA) to quantify protein concentration. MDA levels were then normalized to total protein concentration. During measurement of lipid peroxidation, the purple color in the reaction comes from the reaction of thiobarbituric acid (TBA). MDA was measured by spectrophotometry at 532 nm with respect to the blank solution. We used the same procedure to lyse the cells and determine the protein concentration in the following assays unless otherwise indicated.

### 5.14. Measurement of Intracellular ROS

BSMCs were seeded in 24-well tissue plates for 24 h, then treated with different concentration of β-HB in the medium with or without 10% FBS for 12 h with or without NAC (1.0 mM) or BAPTA/AM(15 µM). The fluorescent probe 20, 70-dichlorofluorescein diacetate (DCF-DA) was used to monitor intracellular accumulation of ROS. For this purpose, 1 ml of DCFH-DA solution (10 µM) was added to the 24-well tissue plates with BSMCs, and plates were incubated at 37°C for 20 min. Cells were then washed three times with PBS, and fluorescence intensity was measured with a Olympus Fluoview-10 confocal microscope with excitation and emission wavelengths of 488 nm and 525 nm, respectively.

### 5.15. Statistical Analysis

Statistical analyses were performed using SPSS software (ver. 13 for Windows; SPSS Inc., Chicago, IL, USA). Significances between groups were determined by one-way analysis of variance (ANOVA), and a Student’s T-test was performed. All values are expressed as the means ± S.E.M. Statistical significances were defined as P<0.05 (* or #P<0.05, ** or ##P<0.01 and *** or ###P<0.001).
